# The Association of Pioglitazone and Urinary Tract Disease in Type 2 Diabetic Taiwanese: Bladder Cancer and Chronic Kidney Disease

**DOI:** 10.1371/journal.pone.0085479

**Published:** 2014-01-10

**Authors:** Mei-Yueh Lee, Pi-Jung Hsiao, Yi-Hsin Yang, Kun-Der Lin, Shyi-Jang Shin

**Affiliations:** 1 Department of Internal Medicine, Kaohsiung Municipal Hsiao-Kang Hospital, Kaohsiung, Taiwan; 2 Division of Endocrinology and Metabolism, Department of Internal Medicine, Kaohsiung Medical University Hospital, Kaohsiung, Taiwan; 3 Graduate Institute of Medicine, Kaohsiung Medical University, Kaohsiung, Taiwan; 4 Graduate Institute of Medical Genetics, Kaohsiung Medical University, Kaohsiung, Taiwan; 5 Statistical Analysis Laboratory, Department of Clinical Research, Kaohsiung Medical University Hospital, Kaohsiung, Taiwan; National Taiwan University, Taiwan

## Abstract

**Objective:**

Although studies have shown an association between pioglitazone and bladder cancer, the associated factors have not been identified. The aim of this study was to investigate the factors that may link pioglitazone to bladder cancer.

**Materials and Methods:**

In total, 34,970 study subjects were identified from the National Health Insurance Research Database in 2003 with follow-up from 2005 to 2009. The demographic characteristics of patients who had used and had never used pioglitazone, including age, sex, diabetes duration, urinary tract disease, nephropathy, bladder cancer, and cumulative dose and duration of pioglitazone therapy, were analyzed using the χ2 test. Cox proportional hazard regression models were used to determine the independent effects of pioglitazone on bladder cancer and newly developed chronic kidney disease.

**Results:**

Among 3,497 ever users and 31,473 never users of pioglitazone, the respective incident cases of bladder cancer were 12 (0.4%) and 72 (0.2%), and for newly developed chronic kidney disease 245 (8.1%) and 663 (2.3%), respectively. Ever use of pioglitazone [1.59(1.32–1.91)], cumulative dose of pioglitazone <10,500 mg [1.69 (1.37–2.01)] and >10,500 mg [1.34 (1.04–1.73)], and duration of therapy <12 months [1.68 (1.36–2.08)] and >12 months [1.39 (1.09–1.76)] were associated with the development of chronic kidney disease.

**Conclusions:**

There was no association of pioglitazone use with bladder cancer development, however, there was an association with an increased risk of newly developed chronic kidney disease.

## Introduction

Peroxisome proliferator-activated receptors (PPARs) are ligand-activated transcription factors which belong to the nuclear receptor superfamily [1]. PPARγ1 is expressed in the heart, skeletal muscles, kidneys, pancreas, and some epithelial tissues such as the urothelium and intestine. In comparison, PPARγ2 is expressed exclusively in adipose tissue and induces adipocyte differentiation as well as being involved in the control of inflammatory reactions and in glucose metabolism through enhanced insulin sensitivity [1], [2]. Since urothelial cells have PPARγ receptors, it has been suggested that a direct effect of the agonist on the urothelial receptor might be the cause of bladder carcinogenesis via these non-DNA reactive agents; further, this effect may be caused by both PPARγ and PPARα agonists via an interaction between their receptors [3]. However, some studies have reported that PPARγ agonists can also inhibit cell proliferation and induce differentiation in various cancer cell lines, such as human urothelial carcinoma [1],[4], rather than increasing cell proliferation as would be expected for a non-DNA reactive chemical's carcinogenic mode of action. Two hypotheses have been formulated regarding urothelial carcinogenesis by these agents. The first involves a direct effect of the agents on the urothelial PPARγ receptors [5]. The second suggests an indirect effect of the agents, which does not specifically target the PPARγ receptors in the urothelium but instead involves physiological or pharmacological fluid changes and the modification of renal function and the altering of fluid dynamics. This then leads to significant changes in urine composition, and in turn results in the formation of various types of urinary solids (precipitate, microcrystals, calculi). These solids are known to be irritative and toxic to the urothelium, especially in rats, and have been observed to cause sustained regenerative proliferation and ultimately to the induction of bladder (urothelial) tumors [6]. Pioglitazone is a thiazolidinedione PPARγ ligand used in the treatment of type 2 diabetes, a disease known to increase the risk of cancer. Several studies have suggested an increased risk of bladder cancer with exposure to pioglitazone [7]–[9]. However, these reports have come from only Western countries, the findings have been inconsistent, and the factors related to this association were not identified. This study is the first to represent an Asian ethnic group and to analyze in detail the risk of bladder cancer with the use of pioglitazone. In this study, we aimed to investigate the possible association of pioglitazone and bladder cancer via the mechanisms in the aforementioned hypotheses.

## Materials and Methods

### Setting

This study use data from the National Health Insurance Research Database (NHIRD), published by the National Health Research Institute (NHI) in Taiwan, which includes data for 1,000,000 randomly selected subjects who were followed from 1998 to 2009. The NHI program was implemented in Taiwan in 1995 and offers a comprehensive, unified, and universal health insurance program to all citizens, including those who have established a registered domicile for at least 4 months in the Taiwan area. The coverage provides outpatient services, inpatient care, Chinese medicine, dental care, childbirth, physical therapy, preventive health care, home care, and rehabilitation for chronic mental illnesses. The coverage rate was 96.16% of the whole the population in 2000 rising to 99% at the end of 2004. The NHI medical claims database includes data on ambulatory care, hospital inpatient care, dental services, and prescription drugs.

### Study population

The entry date of 2003 was selected because pioglitazone was first marketed in Taiwan in 2002. There were no statistically significant differences in age, sex, and average insured payroll-related amount among the enrollees. The diagnosis coding of the NHI program in Taiwan is done according to the International Classification of Diseases, 9^th^ Revision, Clinical Modification (ICD-9-CM) diagnostic criteria. All patients with type 2 diabetes (ICD-9-CM code 250.1–250.9) were followed until the end of 2009. Data on the occurrence of bladder cancer during the study follow-up period (2005 to 2009) were obtained from the same database. Bladder cancer cases were identified according to ICD-9-CM code 188 and were confirmed by the issuance of catastrophic illness cards. After excluding individuals who died or had diabetes after the occurrence of bladder cancer, 34,970 patients with type 2 diabetes were recruited.

Variables determined before entry included age, sex, diabetes duration, newly diagnosed type 2 diabetes at entry, other diabetes medications including sulfonylurea, metformin, acarbose, meglitinides, insulin, bladder cancer, urinary tract disease (ICD-9-CM codes 590-599) and nephropathy (ICD-9-CM codes 580–589). The patients prescribed with pioglitazone before entry were defined as ever users, and those who had never used pioglitazone as never users. Dose responsive parameters including a cumulative dose of less than and more than 10,500 mg, and duration of therapy of less than and more than 12 months, were included. The cutoff criteria of a 10,500 mg dose and a 12-month duration of therapy were established in previous studies [7]–[9].

### Statistical analysis

All data processing and statistical analyses were performed with Statistical Analysis Software (SAS), version 9.1 (SAS Institute, Cary, NC, USA). Chi-square tests were used to analyze the differences in demographic characteristics between the ever users and never users of pioglitazone, which included age, sex, diabetes duration, newly diagnosed type 2 diabetes at entry, other diabetes medications including sulfonylurea, metformin, acarbose, meglitinides, insulin, bladder cancer, urinary tract disease and nephropathy. Cox regression analysis was used to calculate hazard ratios (HRs) of bladder cancer and newly developed chronic kidney disease with pioglitazone use. The confounders of cumulative dose, duration of therapy, urinary tract disease and nephropathy were adjusted by sex, age, duration of diabetes, other diabetes medications, income (monthly income <NT$20,000 and >NT$20,000) and residential area. A P value of less than 0.05 was considered to be statistically significant.

## Results

A total of 34,970 patients with type 2 diabetes were included, with 3,497 ever users and 31,473 never users of pioglitazone. The ever users were more likely to be aged ≧ 60 years, female, lived in Northern counties with monthly income of <20,000 NTD and with a diabetes duration of more than 36 months. Only 69 (2.0%) newly diagnosed type 2 diabetic patients started with pioglitazone as the initial treatment. Most of the ever users had a cumulative dose of ≦ 10,500 mg and duration therapy of ≦ 12 months, but were more likely to have been treated with sulfonylureas, metformin, acarbose, meglitinides and insulin either before, along with, or after pioglitazone. During the follow-up period, there were 84 cases of newly diagnosed bladder cancer (12 ever users and 72 never users). The incidence of nephritis (1.4% vs. 1.0%), chronic kidney disease (7.3% vs. 3.3%), newly developed chronic kidney disease (8.1% vs. 2.3%), hydronephrosis (3.6% vs. 2.5%), calculus of the kidney and ureter (2.7% vs. 1.9%), and other disorders of the urethra and urinary tract (2.4% vs. 1.4%) were higher in the ever users than in the never users (Table 1).

**Table 1:** Demographics of the study cohort according to ever use of pioglitazone.

	Ever use of Pioglitazone N (%)	Never use of Pioglitazone N (%)	P-value
N	3,497	31,473	
Age (years)			
20–29	24 (0.7)	216 (0.7)	
30–39	47 (1.3)	423 (1.3)	
40–49	283 (8.1)	2,547 (8.1)	
50–59	836 (23.9)	7,524 (23.9)	
>60	2,307 (66.0)	20,763 (66.0)	
Sex (female)	1,835 (52.5)	16,515 (52.5)	
Income			
Unemployed	1129 (32.3)	9,167 (29.1)	<.0001
<20,000 NTD	1663 (47.6)	16,072 (51.1)	
>20,000 NTD	705 (20.2)	6,234 (19.8)	
Residence			
Metropolitan	1036 (29.6)	7,851 (24.9)	<.0001
Northern Cities	222 (6.3)	2,441 (7.8)	
Southern Cities	94 (2.7)	1,331 (4.2)	
Northern Counties	1374 (39.3)	13,420 (42.6)	
Southern Counties	763 (21.8)	6,315 (20.1)	
Nephritis	50 (1.4)	307 (1.0)	0.0112
Chronic kidney disease	256 (7.3)	1,038 (3.3)	<.0001
Infection of kidney	25 (0.7)	172 (0.5)	0.2069
Hydronephrosis	125 (3.6)	790 (2.5)	0.0002
Calculus of kidney and ureter	94 (2.7)	595 (1.9)	0.0013
Cystitis	113 (3.2)	1,059 (3.4)	0.6774
Other disorder of urethra and urinary tract	84 (2.4)	455 (1.4)	<.0001
Other diabetes medication			
Sulfonylureas	1,481 (42.4)	2,916 (9.3)	<.0001
Metformin	1,344 (38.4)	2,631 (8.4)	<.0001
Acarbose	757 (21.6)	729 (2.3)	<.0001
Meglitinides	546 (15.6)	615 (2.0)	<.0001
Insulin	2,918 (83.4)	24,770 (78.7)	<.0001
Newly diagnosed diabetes at the start of follow-up	69 (2.0)	24,796 (78.8)	<.0001
Duration of diabetes (months)			
<36	667 (19.1)	2,401 (7.6)	<.0001
>36	2,761 (79.0)	4,276 (13.6)	
Cumulative dose (mg)			
<10,500	2,313 (66.1)	N/A	
>10,500	1,184 (33.9)	N/A	
Duration of therapy (months)			
<12	2,145 (61.3)	N/A	
>12	1,352 (38.7)	N/A	
Bladder cancer	12 (0.4)	72 (0.2)	0.1761
Newly developed chronic kidney disease	245 (8.1)	663 (2.3)	<.0001

**Figure 1:**
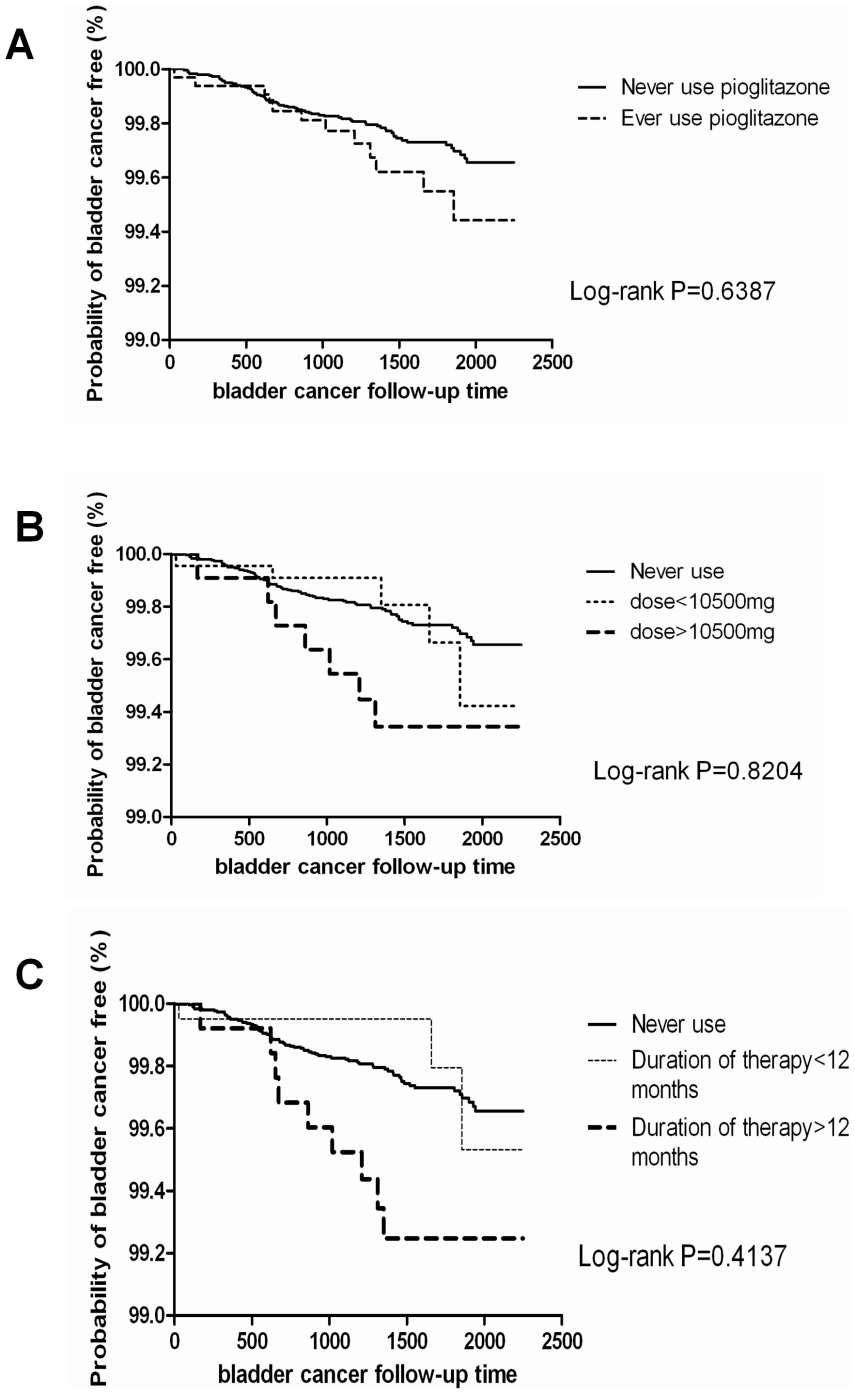
Disease free survival of bladder cancer. (A).never use and ever use of pioglitazone. (B).never use, cumulative dose <10500 mg and >10500 mg of pioglitazone. (C).never use, duration of therapy <12 months and >12 months of pioglitazone.

In the analysis of the association bladder cancer, the risk of bladder cancer increased with a cumulative dose of pioglitazone of more than 10,500 mg [HR 2.21 (95% CI 1.01–4.80), P = 0.0461], and duration of therapy of pioglitazone more than 12 months [HR 2.52 (95% CI 1.26–5.05), P = 0.0090], newly developed chronic kidney disease [HR 7.50 (95% CI 4.26–13.21), P<0.0001], and calculus of the kidney and ureter [HR 3.41 (95% CI 1.38–8.41), P = 0.0078]. After fully adjusting for sex, age, duration of diabetes, other diabetes medications, income and residential area, nephritis, chronic kidney disease, kidney infections, hydronephrosis, calculus of the lower urinary tract, calculus of the kidneys and ureter, cystitis, other disorders of the urethra and urinary tract, newly developed chronic kidney disease, hypertension and hyperlipidemia, no significant associations were found between the ever users of pioglitazone, cumulative dose and duration of therapy with the risk of bladder cancer (Table 2, Figure 1). However, there were significant associations between newly developed chronic kidney disease [HR 5.45 (95% CI 2.99–9.93), P<0.0001] and calculus of the kidney and ureter [HR 2.75 (95% CI 1.01–7.52), P = 0.484] with the risk of bladder cancer (Table 2).

**Table 2:** Hazard ratio of bladder cancer.

Bladder Cancer	Univariate		Multivariate*	
	HR (95% CI)	*P*	HR (95% CI)	*P*
Pioglitazone use				
Never use (n = 72)	1		1	
Ever use (n = 12)	1.52 (0.83–2.80)	0.1787	1.03 (0.45–2.35)	0.9452
Cumulative dose of pioglitazone (mg)				
Never use (n = 72)	1		1	
<10,500 (n = 5)	1.03 (0.42–2.55)	0.9530	0.84 (0.30–2.35)	0.7429
>10,500 (n = 7)	2.21 (1.01–4.80)	0.0461	1.27 (0.45–3.61)	0.6540
Duration of therapy of pioglitazone (months)				
Never use (n = 72)	1		1	
<12 (n = 3)	0.67 (0.21–2.13)	0.4978	0.56 (0.16–1.95)	0.3605
>12 (n = 9)	2.52 (1.26–5.05)	0.0090	1.58 (0.62–4.02)	0.3355
Newly developed chronic kidney disease				
No	1		1	
Yes	7.50 (4.26–13.21)	<.0001	5.45 (2.99–9.93)	<.0001
Calculus of kidney and ureter				
No	1		1	
Yes	3.41 (1.78–8.41)	0.0078	2.75 (1.04–7.52)	0.0484

Adjusted by sex, age, duration of diabetes, other diabetes medications, income (monthly income <NT$20,000; monthly income >NT$20,000), residential area, nephritis, chronic kidney disease, kidney infections, hydronephrosis, calculus of the lower urinary tract, cystitis, other disorders of the urethra and urinary tract, hypertension and hyperlipidemia. The adjusted HR of newly developed chronic kidney disease and calculus of the kidney and ureter were computed with the indicated variable of pioglitazone use.

**Figure 2:**
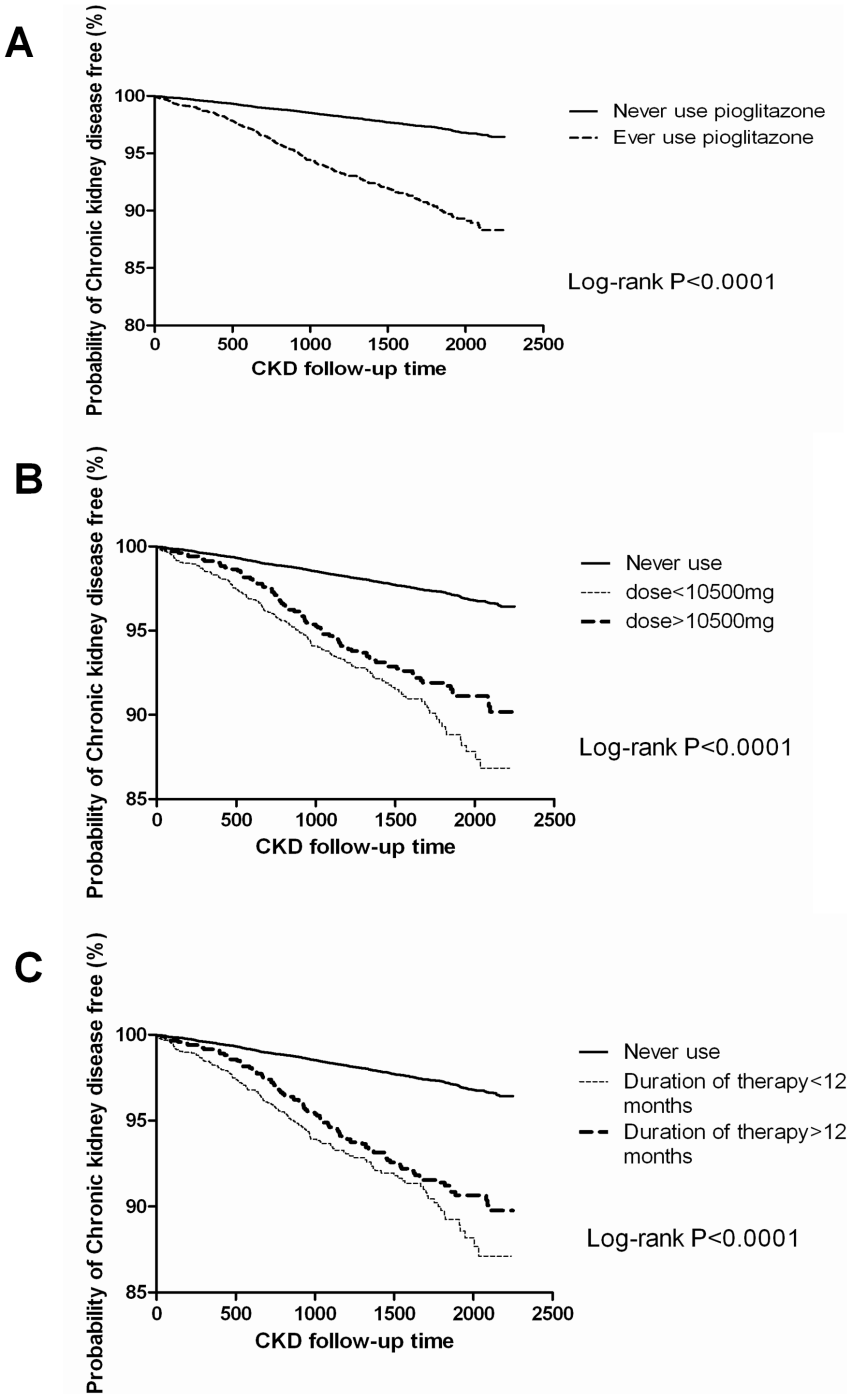
Disease free survival of newly developed chronic kidney disease after use of pioglitazone. (A).never use and ever use of pioglitazone. (B). never use, cumulative dose <10500 mg and >10500 mg of pioglitazone. (C). never use, duration of therapy <12 months and >12 months of pioglitazone.

With regards to the association of newly developed chronic kidney disease, there were significantly increased risks of newly developed chronic kidney disease in the pioglitazone ever users [HR 1.66 (95% CI 1.37–2.01), P<0.0001), cumulative dose of pioglitazone ≦10,500 mg [HR 1.81 (95% CI 1.46–2.25), P<0.0001] and ≧ 10,500 mg [HR 1.39 (95% CI 1.07–1.80), P = 0.0134], duration of therapy of pioglitazone ≦ 12 months [HR 1.79 (95% CI 1.44–2.24), P<0.0001] and ≧ 12 months [HR 1.46 (95% CI 1.14–1.86), P = 0.0027], hypertension [HR 4.01 (95% CI 3.47–4.63), P<0.0001], hyperlipidemia [HR 2.44 (95% CI 2.14–2.78), P<0.0001] and the diabetes medication, meglitinides [HR 4.03 (95% CI 3.27–4.96), P<0.0001]. After fully adjusting for age, sex, duration of diabetes, income and residential area, nephritis, chronic kidney disease, kidney infections, hydronephrosis, calculus of the lower urinary tract, calculus of the kidney and ureter, cystitis, other disorders of the urethra and urinary tract, and other diabetes medication including sulfonylureas, metformin, acarbose, rosiglitazone and insulin, there were significantly increased risks of newly developed chronic kidney disease with hypertension [HR 2.14 (95% CI 1.82–2.52), P<0.0001], hyperlipidemia [HR 1.26 (95% CI 1.09–1.45), P = 0.0020], use of meglitinides [HR 1.68 (95% CI 1.14–2.48), P = 0.0092], and pioglitazone ever users [HR 1.59 (95% CI 1.32–1.91), P<0.0001]. However, there were slight decreases in the adjusted hazard ratios for cumulative dose of pioglitazone ≧ 10,500 mg [HR 1.34 (95% CI 1.04–1.73), P = 0.0232] and ≦ 10,500 mg [HR 1.69 (95% CI 1.37–2.07), P<0.0001]. Decreases in the adjusted hazard ratio of newly developed chronic kidney disease were also observed with duration of therapy ≧ 12 months [HR 1.39 (95% CI 1.09–1.76), P = 0.0072] and ≦12 months [HR 1.68 (95% CI 1.36–2.08), P<0.0001]. Table 3 (see also Figure 2).

**Table 3:** Hazard ratios of newly developed chronic kidney disease.

	Univariate		Multivariate*	
Pioglitazone use	HR (95% CI)	*P*	HR (95% CI)	*P*
Never use (n = 663)	1		1	
Ever use (n = 245)	1.66(1.37–2.01)	<.0001	1.59(1.32–1.91)	<.0001
Cumulative dose of pioglitazone (mg)				
Never use (n = 663)	1		1	
<10,500 (n = 160)	1.81(1.46–2.25)	<.0001	1.69(1.37–2.07)	<.0001
>10,500 (n = 85)	1.39(1.07–1.80)	0.0134	1.34(1.04–1.73)	0.0232
Duration of therapy of pioglitazone (months)				
Never use (n = 663)				
<12 (n = 144)	1.79(1.44–2.24)	<.0001	1.68(1.36–2.08)	<.0001
>12 (n = 101)	1.46(1.14–1.86)	0.0027	1.39(1.09–1.76)	0.0072
Hypertension				
No	1		1	
Yes	4.01(3.47–4.63)	<.0001	2.14(1.82–2.52)	<.0001
Hyperlipidemia				
No	1		1	
Yes	2.44(2.14–2.78)	<.0001	1.26(1.09–1.45)	0.0020
Other diabetes medication				
Meglitinides				
No	1		1	
Yes	4.03 (3.27–4.96)	<.0001	1.68 (1.14–2.48)	0.0092

Adjusted by age, sex, diabetes duration, income (monthly income <NT$20,000; monthly income >NT$20,000) residential area, nephritis, chronic kidney disease, kidney infections, hydronephrosis, calculus of the lower urinary tract, calculus of the kidney and ureter, cystitis, other disorders of the urethra and urinary tract, other diabetes medication including sulfonylureas, metformin, acarbose, rosiglitazone and insulin. The adjusted HR of hypertension, hyperlipidemia and other diabetes medication were computed with the indicated variable of pioglitazone use.

The adjusted hazard ratios in Table 2 and 3 were computed by multiple Cox regression with one of the pioglitazone usage variable at a time. Since the hazard ratios of cumulative dose and duration of therapy of pioglitazone were very similar, therefore only one set was listed in table

## Discussion

We observed no association of bladder cancer with the ever users, cumulative dose and therapy duration of pioglitazone, but a positive association with newly developed chronic kidney disease. Using pioglitazone and/or meglitinides as the initial treatment increased the risk of chronic kidney disease, and also for those with a history of hypertension and hyperlipidemia.

Although several studies have suggested an increased risk of bladder cancer with exposure to pioglitazone [7]–[9], the factors related to this association have not been identified. In the current study, we found that urinary tract diseases, such as calculus of the kidney and ureter, and chronic kidney diseases increased the risk of developing bladder cancer rather than the use of pioglitazone, for which we found no association with the development of bladder cancer in contrast to previous studies.

PPARγ and combined agonists have been shown to produce urothelial tumors in rats as well as suspected urothelial changes in monkeys and possibly in dogs [10]. These effects are either due to a direct effect on PPARγ in the urothelium or a pharmacologically based indirect mechanism in the animals leading to alterations in urine composition. A characteristic effect of PPARγ and dual agonists is the induction of an enlarged cardiac size and accumulation of body water. Physiological or pharmacological effects involving fluid changes modify renal function, alter fluid dynamics, and lead to significant changes in urine composition, which result in the formation of various types of urinary solids (precipitate, microcrystals, calculi). These these urinary solids are known to be irritative and toxic to the urothelium, especially in rats, and have been observed to lead to sustained regenerative proliferation and ultimately to the induction of bladder (urothelial) tumors [6]. This effect has been reported to be greater in male than in female rats, and greater in rats than in mice, however much less likely in primates such as humans [11]. In the current study, the associations of calculus of the kidney and ureter and bladder cancer were not related to pioglitazone. The Food and Drug Administration has accepted an increase in heart size as a determinant for a maximum tolerated dose for this class of drugs [12]. This effect has been reported to be dose-dependent and a pharmacologically-based response to these agents [13]–[15]. The indirect pharmacological mode of action produced by PPAR agonists is not unique to the urothelium, and indirect effects have also been seen with PPARγ agonists in the production of rat pancreatic acinar cell tumors and, to some extent, rat testicular Leydig cell neoplasms. However, these neoplasms do not appear to be relevant to humans [16]. A variety of other indirect mechanisms have been identified with other classes of chemicals operating through other modes of action. However, a direct effect on urothelial PPARγ receptors as the cause of the carcinogenic response is highly unlikely for a variety of reasons, most notably the fact that the biological effect of these agonists on the urothelium is to inhibit proliferation rather than to increase the rate of proliferation expected for a carcinogen [17].

Chronic kidney disease is a proinflammatory state [18]–[20], and there is now emerging evidence of an association between chronic inflammation and the risk of cancer [21]. Recently, an association between elevated albumin-to-creatinine ratio and cancer was reported in a longitudinal population-based study of older individuals [22]. Observational studies have also suggested an increased cancer risk in people with early-stage chronic kidney disease before requiring dialysis or transplantation [23]. It has been reported that for every 10-ml/min decrement in estimated glomerular filtration rate (eGFR), the risk of cancer increases by 29% (adjusted HR 1.29; 95% CI 1.10 to 1.53), with the greatest risk at an eGFR ≦40 ml/min per 1.73 m2 (adjusted HR 3.01; 95% CI 1.72 to 5.27), especially in the risk for lung and urinary tract cancers but not prostate cancer in men with chronic kidney disease [24]. In the current study, chronic kidney disease was found to increase the risk of bladder cancer. Chronic kidney disease is associated with significant morbidity and premature death. Markers of inflammation such as white blood cell count have also been associated with an increased risk for cancer mortality in the general population [25]–[27].

Among oral antihyperglycemic agents, sulfonylureas (glyburide, gliclazide, glipizide, glibenclamide, tolbutamide, and chlorpropamide) have been reported to have increased potency as renal function decreases, and are contraindicated in patients with severe renal failure [28]. Similarly, α-glucosidase inhibitors (acarbose and miglitol) are also contraindicated in patients with renal failure, whereas the non-sulfonylurea insulin secretagogues repaglinide and nateglinide can be used in patients with renal failure without dose adjustments [29]. In the absence of contraindications, metformin is preferred over other agents due to equal potency and a low risk of hypoglycemia, and also as it causes less weight gain compared with insulin secretagogues. In obese patients, metformin has shown strong clinical evidence of reduced microvascular and macrovascular outcomes [30]. Metformin is contraindicated in patients with renal failure because of the associated risk of lactic acidosis [31]. In the presence of contraindications or intolerance to metformin or when metformin alone does not result in optimal control, thiazolidinediones should be used [30]. Although the metabolism of thiazolidinediones is unaffected by renal failure, they must be used with caution in this context because of their volume retaining effect with a risk of heart failure [32]. Therefore, in chronic renal failure, the oral agents that can be used include the insulin secretagogues repaglinide and nateglinide and the thiazolidinediones (rosiglitazone and pioglitazone), although they should be used with caution. Insulin can also be used safely in patients with renal failure [30]. However, in the current study, we found that pioglitazone, repaglinide and nateglinide increased the risk of developing chronic kidney disease.

Cardiovascular complications and mortality have been reported to be increased in patients with chronic kidney disease independent of traditional risk factors such as diabetes, hypertension, and dyslipidemia [33]–[35]. Post hoc analysis from the PROspective pioglitAzone Clinical Trial In macroVascular Events (PROactive) investigated the relationship between chronic kidney disease and the incidence of cardiovascular disease in patients with diabetes and documented macrovascular disease, as well as the effects of pioglitazone treatment on recurrent cardiovascular disease, and found that pioglitazone was more effective than placebo in reducing the rate of both primary and secondary composite end points in the patients with chronic kidney disease. There was a nonsignificant 25% risk reduction for pioglitazone relative to the placebo for the primary end point (95% CI 0.55 to 1.03) and a significant 34% relative risk reduction for the secondary end point (95% CI 0.45 to 0.98). The yearly declines in GFR (0.9 ml/min per 1.73 m2 with placebo and 1.8 ml/min per 1.73 m2 with pioglitazone) in one study [36] were considerably lower than the decline of 3 to 4 ml/min per 1.73 m2 observed in patients with diabetes in previous studies [37],[38], and were more in the range of the GFR decrease found in an aging healthy population (1 ml/min per 1.73 m2/year) [39]. In the current study, the use of pioglitazone was found to increase the risk of chronic kidney disease.

There are several strengths to this study. It is a prospective follow-up study of a very large cohort of diabetic and cancer patients with a high likelihood of the correct diagnosis of cancer and diabetes by use of the computerized data files for each individual from the NHIRD. Nevertheless, there are some limitations including the lack of actual measurements for confounders such as biochemical data, obesity, tobacco smoking, occupational exposure, lifestyle and diet. Instead of tobacco smoking history, we thus analyzed the association of bladder cancer, calculus of the kidney and ureter, and newly developed chronic kidney disease with pioglitazone separately by gender, however no significant differences were found. We also included the residential area and income as confounders instead of occupational exposure, lifestyle and diet, and used hyperlipidemia for obesity. In addition, the relationship between the duration of therapy and cumulative dose of pioglitazone with the possibility of renoprotection cannot be proven further due to the short historical use of pioglitazone in Taiwan. Further studies are warranted to elucidate this relationship.

In summary, there was no significant increases in the incidence of bladder cancer in the ever users of pioglitazone, therapy duration and cumulative dose of pioglitazone. However, pioglitazone use increased the risk of developing chronic kidney disease,
